# Seasonal Influence on Volatile Organic Compounds from Flowers and Leaves of *Lepechinia mutica* Extracted by SPME-GC-MS

**DOI:** 10.3390/plants14193103

**Published:** 2025-10-09

**Authors:** James Calva, Dayanna Suquilanda, Ángel Benítez, Chabaco Armijos, Jorge Ramírez

**Affiliations:** 1 Departamento de Química, Universidad Técnica Particular de Loja, Calle Paris s/n y Praga, Loja 110107, Ecuador; cparmijos@utpl.edu.ec (C.A.); jyramirez@utpl.edu.ec (J.R.); 2 Carrera Ingeniería Química, Universidad Técnica Particular de Loja, Loja 1101608, Ecuador; dpsuquilanda@utpl.edu.ec; 3 Biodiversidad de Ecosistemas Tropicales-BIETROP, Herbario HUTPL, Departamento de Ciencias Biológicas y Agropecuarias, Universidad Técnica Particular de Loja (UTPL), San Cayetano s/n, Loja 1101608, Ecuador; arbenitez@utpl.edu.ec

**Keywords:** *Lepechinia mutica*, SPME-GC-MS, volatile compounds, PCA analysis

## Abstract

*Lepechinia mutica,* an endemic species of the Ecuadorian Andes, was studied to identify the seasonal variation in volatile organic compounds emitted from leaves and flowers in winter and summer using solid-phase microextraction–gas chromatography–mass spectrometry (SPME-GC-MS). A total of 101 and 100 volatile compounds were identified in flowers and leaves, respectively. The main compounds in flowers were β-phellandrene (7.81–17.74%), dictamnol (3.57–31.89%) and 9-epi-(E)-caryophyllene (3.93–14.37%), while in the leaves, they were dictamnol (9.85–34.64%), (Z)-β-ocimene (1.24–29.24%) and δ-3-carene (1.14–11.51%). This is the first report of enantiomeric separation in *L. mutica* using a capillary column with 2,3-diethyldecyl-6-tert-butyl-dimethylsilyl-β-cyclodextrin, revealing three enantiomerically pure compounds as (*S*)-(-)-β-pinene, (1*S*,3*R*)-(+)-δ-3-carene and (*S*)-(+)-linalool, while (+) (-) α-pinene, (+) (-) δ-cadinene and (+) (-) α-muurolene were found as racemic mixtures. Principal component analysis confirmed distinct chemical profiles between plant parts and seasons. This result has important implications for the future highlighting its potential as a source of seasonally variables components with applications in fragrance and phytotherapy.

## 1. Introduction

Climate is a primary driver of plant ecosystem dynamics, directly regulating key physiological processes, including photosynthesis, transpiration, nutrient cycling and the biosynthesis of primary and secondary metabolites [1]. While not essential for basic growth or survival, secondary metabolites contribute to the fundamental metabolic functions necessary for plant survival; they play an essential role in enhancing plant performance under biotic and abiotic stress conditions, such as pathogen attacks or nutrient limitation [2,3]. These compounds have been associated with protective and defensive functions, including antioxidant activity, as well as antibiotic, insecticidal and herbicidal properties against external aggressions [4]. Essential oils are complex mixtures of volatile secondary metabolites; they are derived from plants and are highly responsive to both intrinsic (e.g., phenology) and extrinsic (e.g., temperature, UV, drought) factors, leading to dynamic seasonal shifts in their chemical composition [5,6]. These variations directly affect the biological activity of plant extracts, highlighting the importance of understanding how environmental and physiological cues together influence essential oil profiles for successful phytochemical discovery and pharmacological application [7].

The Lamiaceae family, commonly known as the mint or deadnettle family, comprises approximately 236 genera and over 7000 species of herbs, shrubs and trees [8]. It is widely recognized for its rich diversity of essential oils and volatile compounds which confer distinctive aromatic and biological properties [9]. Within this family, Lepechinia is distinguished by unique fragrance and potential as a source of bioactive compounds exhibiting antimicrobial, antioxidant, anti-inflammatory and neuroprotective activities [10]. *Lepechinia mutica* is an endemic plant species native to Ecuador, commonly found in cloud forests, páramos, tropical forests and grasslands at elevations between 2200 and 3400 m a.s.l. [11,12]. Traditionally, it is used in folk medicine to treat various ailments, headache and nervous disorders and as antiseptic remedy [13,14,15]. However, the limitation of scientific research on this species makes it a promising candidate for further investigation into its chemical composition, aromatic profile and seasonal variability. The analysis of its volatile organic compounds using solid-phase microextraction coupled with gas chromatography–mass spectrometry (SPME-GC-MS) could provide significant contributions to the discovery of novel chemical entities and the development of new pharmaceuticals [16].

We hypothesized that seasonal changes in abiotic stresses (e.g., temperature, ultraviolet radiation and drought) would lead to distinct volatile profiles in leaves and flowers. Chemical characterization of volatile organic compounds in the leaves and flowers of *L. mutica* is essential to understand its aromatic profile and seasonal variation. Volatile compounds are small, lipophilic molecules that play critical roles in plant environment interactions, including pollinator attraction, defense against pathogens and herbivores and adaptation to changing environmental conditions [17]. While previous studies on Lepechinia spp. have identified sesquiterpenes, monoterpenes and diterpenes as the main constituents [18], our SPME-GC-MS approach, unlike the hydrodistillation method employed by Guzmán et al. (2022), which identified shyobunol, δ-3-carene and globulol [19], revealed dictamnol to be the dominant sesquiterpene. This highlights the critical role of extraction methodology in shaping observed chemical profiles.

This study employs HS-SPME-GC-MS to characterize seasonal variation in the volatile organic compounds from leaves and flowers of *L. mutica*. Extractions were performed during winter and summer with chemical profiles analyzed using a non-polar column. Additionally, preliminary enantioselective analyzes of the leaves and flowers are reported. It is expected that valuable data for the conservation and sustainable utilization of this endemic species will be provided by this work as well as a greater understanding of its chemical composition.

## 2. Results

### 2.1. Chemical Composition

The chemical composition variability of volatile compounds in flowers of *L. mutica* is presented in Table 1. A total of 101 compounds were identified in the winter and summer flower samples. The major constituents with the ranges included β-phellandrene (7.81–17.74%), dictamnol (3.57–31.89%), 9-epi-(E)-caryophyllene (3.93–14.37%) and bicyclogermacrene (2.4–9.74%), among others. A predominance of sesquiterpenes hydrocarbons was observed, accounting a range of 41.15–54.9%, followed by hydrocarbon monoterpenes 17.69–37% while the oxygenated sesquiterpenes were present at 3.9–8.64% (Figures S1–S6).

Chemical composition of *L. mutica* leaves revealed the presence of 100 compounds. The major constituents were dictamnol (26.45% ± 14.37%), (Z)-β-ocimene (19.05% ± 8.93%), α-cuprenene (7.60% ± 2.81%), camphene (4.81% ± 0.69%) and linalool isovalerate (4%). (79% ± 1.19%), α-zingiberene (4.30% ± 3.73%), α-pinene (3.45% ± 0.43%), trans-β-guaiene (3.11% ± 0.48%), *p*-mentha-1(7),8-diene (2.21% ± 0.94%) and myrcene (2.07% ± 1.77%). The main compound groups identified were hydrocarbon monoterpenes (47.41%, 27.85% and 25.30%), hydrocarbon sesquiterpenes (42.69%, 61.26% and 63.12%) and oxygenated sesquiterpenes (13.37%, 8.55% and 13.17%) and oxygenated monoterpenes were present in lower proportions with 1.77%, 1.08% and 1.29%, respectively (Table 2) (Figures S7–S12).

These differences in chemical profiles between seasons could be explained by variations in biosynthetic stages and the physiological needs of the plant. Sesquiterpenes are larger, less volatile molecules compared to monoterpenes and are often associated with protective and defensive functions. This pattern suggests a possible functional adaptation of the plant’s volatile metabolism in response to seasonal environmental conditions (Figure S13).

### 2.2. PCA Analysis

**Figure 1:**
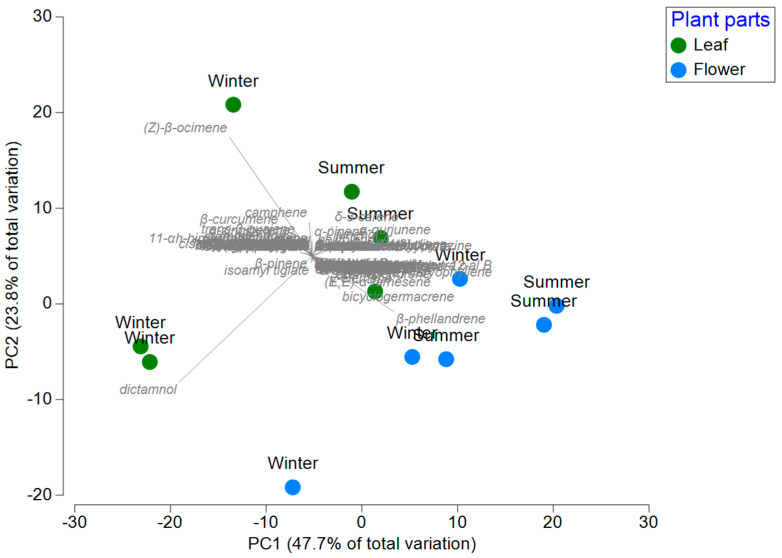
Principal coordinate analysis showing variation between chemical compounds of *L. mutica* (Benth.) in different parts of plants and seasons.

PCoA showed clear segregation by plant part (leaves vs. flowers) and season indicating that chemical composition is primarily structured by season and plants parts (PC1 47.7% and 23.8% variance). Evidence of the results obtained using the corresponding tool was presented in the statistical analysis. It was possible to group all the compounds and highlight those that were predominant in the different seasons of the year. In winter, the predominant compounds were aromadendrene and (Z)-β-ocimene. By contrast, the main compounds identified in summer were dictammol and junenol. The Principal Componet Analysis (PCoA) ordination showed a clear separation between chemical composition of *L. mutica* and parts of plants and season (Figure 1).

PERMANOVA analyses showed that chemical composition of *L. mutica* was structured according to the parts of plants and season, and a large component of variation was associated with the parts of plants with 22.4% of explained variance in chemical composition of *L. mutica*. This was followed by the season, accounting for 14.5% of the explained variance (Table 3). Conversely, the interaction between plant parts (leaves and flowers) and season does not have significant effects (*p* value = 0.074).

Results of pairwise PERMANOVA test between chemical composition of *L. mutica* according to parts of plants (62.78%, *p*-value = 0.001) and season (58.73%, *p* value = 0.002) showed that the highest significant dissimilarity values for chemical composition were associated with parts of plants followed by season.

The SIMPER routine revealed that not all chemical compounds contribute equally to establishing the differences in the parts of plants. We observed that the largest contributions are due to differences in chemical compounds of β-phellandrene (*Z*)-β-ocimene and bicyclogermacrene (Table 4).

### 2.3. Enantioselective Analysis

Enantioselective analysis using a chiral capillary column (2,3-diethyl-6-tert-butyl dimethylsilyl-β-cyclodextrin as a chiral selector) resolved nine enantiomers in *L. mutica* flowers and leaves. Three compound as (*S*)-(-)-β-pinene, (1*S*,3*R*)-(+)-δ-3-carene and (*S*)-(+)-linalool were identified as enantiomerically pure (*e.e.* 100%). The remaining six as (1*R*)-(+)-α-pinene, (1S)-(-)-α-pinene (1S,4S,7*R*)-(+)-δ-cadinene, (1*R*,4*R*,7S)-(–)-δ-cadinene, (6*R*,8S)- (+)-α-muurolene and (6S,8*R*)-(–)-α-muurolene occurred as racemic mixtures (Table 5 and Table 6).

## 3. Discussion

Volatile compounds emitted by flowers and leaves of the endemic species *L. mutica* using SPME-GC-MS revealed its complex chemical profile, highlighting its potential as a source of bioactive compounds. The results showed significant seasonal variations in chemical composition, with these variations depending on the time at which the samples were collected. These variations are likely influenced by environmental factors such as temperature, humidity, solar radiation, drought stress, and ultraviolet-B radiation affect secondary metabolic pathways in plants [22,23,24].

In flowers, the major compounds identified during winter were (*Z*)-β-ocimene, aromadendrene and linalool isovalerate, while the summer samples showed higher levels of 9-epi-(*E*)-caryophyllene and α-gurjunene. These results partially align with those reported by Guzmán et al. [19], who analyzed the essential oil of *L. mutica* obtained via hydrodistillation and identified shyobunol (10.80%), δ-3-carene (8.69%), δ-cadinene (6.96%), globulol (5.91%) and (*E*)-caryophyllene (4.55%). The marked compositional differences likely stem from methodological effects, in the hydrodistillation high temperatures can induce thermal degradation or artifact formation [25], whereas HS-SPME-GC-MS provides a non-destructive, in situ snapshot of the plant’s natural volatile profile, better reflecting true biological emissions.

In leaves, the most abundant compounds in winter were (*Z*)-β-ocimene (18.04 ± 2.43%) and 9-epi-(*E*)-caryophyllene (14.23 ± 0.12%), while in summer were dictamnol (26.45 ± 14.37%) and (*Z*)-β-ocimene (19.05 ± 8.93%). Aromadendrene was the most prevalent compound in both seasons, although its concentration varied significantly between them. For instance, Ramírez et al. [25] identified 78 compounds in the essential oil of *L. mutica*, with hydrocarbon sesquiterpenes and oxygenated monoterpenes being the most abundant groups. However, the results demonstrate considerable variation depending on the collection site and season, suggesting a strong influence of environmental [26,27,28] and phenological factors on secondary metabolite production [29].

Our results indicated that the season is a determining factor in the chemical composition. Similarly, previous studies have pointed out that plants are intimately connected to their environment and are therefore strongly influenced by climatic variables, including seasonal changes [30]. It is consistent with previous studies that have highlighted the influence of environmental and phenological factors on the biosynthesis and accumulation of secondary metabolites in medicinal plants. It is well established that various genetic, ontogenic, morphogenetic, and environmental factors can affect the production of natural products [31,32]. As herbs develop, they progress through predictable stages: seedling, vegetative growth, flowering, fruiting, and senescence, each potentially associated with distinct metabolic profiles [33]. Natural products can be gradually synthesized or upregulated in response to environmental stressors. In this context, it has been demonstrated that temperature, radiation, water availability, and other abiotic factors significantly impact secondary metabolism [34]. Importantly, these variables do not act in isolation but change synergistically throughout the growing season. Therefore, seasonal variation encompasses a set of ecologically relevant and interrelated parameters that collectively shape the chemical phenotype of the plant [35]. Drought and UV radiation, which are key abiotic stressors in the Andean cloud forests where *L. mutica* thrives, can trigger shifts in secondary metabolism by altering phenology, resource allocation and the synthesis of defensive compounds. These physiological responses can directly affect floral scent profiles, potentially reducing pollinator attraction while increasing herbivore deterrence. These trade-offs highlight how environmental pressures act as selective drivers of chemical variability, influencing not only the quantity, but also the enantiomeric composition, of volatiles that are essential for ecological function. This work contributes novel data on the seasonal variability of volatile compounds in *L. mutica*, an aspect that has been scarcely explored to date.

Another relevant finding of this study was the identification and separation of enantiomers, a total of nine enantiomers were successfully separated using chiral gas chromatography. Notably, compounds such as (*S*)-(-)-β-pinene, (1*S*,3*R*)-(+)-δ-3-carene and (*S*)-(+)-linalool were found to be enantiomerically pure. By contrast, racemic mixtures were observed for (+) (-)-α-pinene, (+) (-)-δ-cadinene and (+) (-)-α-muurolene. This is particularly important as enantiomers may exhibit distinct or even opposing, biological activities depending on their spatial configuration [36]. For example, the (+)-α-pinene enantiomer has been shown to have more potent anti-inflammatory effects than its (−) enantiomer [37], highlighting the importance of considering chirality in future pharmacological research.

Furthermore, several of the compounds identified in this study have previously been associated with promising therapeutic properties. In our study, one of the major compounds was Dictamnol; it has been documented to exhibit antimicrobial, anti-inflammatory and cytotoxic activities [38]. Concurrently, (Z)-β-ocimene, a major monoterpene found in the leaves and flowers of the plant, is a well-known generalist pollinator attractant and plays a key role in the plant’s defense against herbivory [39]. Furthermore, molecular docking and simulation studies reveal that (Z)-β-ocimene forms stable interactions with α-glucosidase, suggesting an inhibitory effect that could be relevant to metabolic disorders such as diabetes [40]. (*Z*)-β-ocimene is more prevalent and emitted in greater abundance in floral aromas than its isomer (*E*)-β-ocimene [41].

The seasonal variability observed in the chemical composition suggests that we should time biomass collection according to the desired target. This strategic approach could benefit not only the pharmaceutical and cosmetic industries but also contribute to the conservation of the species by promoting sustainable cultivation practices.

This study provides detailed insights into the chemical profile of *L. mutica*. However, key limitations remain. It would be valuable to investigate the influence of factors such as altitude, soil type, local climatic and land practices (e.g., wild harvesting versus cultivation) on the production of secondary metabolites in this species. There is also an absence of replicated sites across altitudinal gradients and the lack of metabolomic quantification. Another promising area for future research would be to assess the biological activity of individual enantiomers. Given the potential applications of these compounds in health and wellness, it is important to understand how their chiral configuration affects biological activity as this could lead to the development of more selective and effective drugs. Future work will involve multi-site sampling, controlled environmental trials and the targeted quantification of key enantiomers.

## 4. Materials and Methods

### 4.1. Plant Material

The leaves and flowers of *L. mutica* were collected in 2024 during two distinct seasons: winter (February, March and April) and summer (June, July and August). For each sampling period, samples were taken from three independent individuals, with no repeated sampling from the same plants. Each sample (*n* = 3 per month × part) was collected from a different plant in Loja province, South of Ecuador (coordinates: 3°59′59″ S; 79°12′45″ W). Sampling occurred during mean temperature ranges: winter (12–18 °C, RH 75–85%) and summer (16–22 °C, RH 65–75%) and monthly rainfall <50 mm (winter) vs. >200 mm (summer). The species was authenticated by Dr. Jorge Ramírez from the Universidad Técnica Particular de Loja (UTPL). Collection was carried out under official permission from the Ministry of Environment, Water and Ecological Transition (Authorization No. MAATE-ARSFC-2022-2839). A voucher specimen No. 14778 was deposited at the Herbarium of the Universidad Técnica Particular de Loja (HUTPL) for future reference.

### 4.2. Headspace Solid-Phase Microextraction

Samples were collected in the early morning hours and analyzed immediately to minimize chemical degradation. Young leaves and flowers were selected for analysis. Following collection, 5 ± 0.1 g of each sample was weighed using an analytical balance (OHAUS Adventurer™ Pro, model 30061977, OHAUS Corporation, Parsippany, NJ, USA). The weighed material was then placed into a transparent 100 mL glass vial (Boeco). For volatile compound extraction, headspace solid-phase microextraction (HS-SPME) was performed using a Carboxen™/DVB/PDMS fiber (50/30 µm, StableFlex 24 Ga, Manual, 3/pk, gray; SUPELCO Inc., Bellefonte, PA, USA). Once inserted into the vial, the fiber remained in the headspace for a defined period to allow adsorption of volatile compounds before thermal desorption and analysis by GC–MS [42]. The fiber was equilibrated at 40 °C for 30 min (previously determined by an adsorption curve) with RSD < 5% (*n* = 3) for repeatability and r^2^ > 0.99 for linearity (range 0.1–10 µg/mL).

### 4.3. Gas Chromatography–Mass Spectrometry (GC–MS) Analysis

For qualitative analysis, volatile compounds were identified using a Thermo Scientific TRACE 1310 gas chromatograph (Waltham, MA, USA) coupled to a Thermo Scientific ISQ 7000 mass spectrometer (Bartlesville, OK, USA). Ultrapure helium (Indura GC grade, Guayaquil, Ecuador) was used as the carrier gas at a constant flow rate of 1 mL/min. The injection mode was set to split (split ratio 10:1), with an injection temperature of 250 °C. A non-polar DB-5ms column (5% phenyl, 95% dimethylpolysiloxane; 30 m × 0.25 mm i.d., film thickness 0.25 µm) was used under the following temperature program: initial temperature of 40 °C held for 5 min, followed by a first ramp of 3 °C/min to 150 °C, then a second ramp of 5 °C/min to 180 °C, and finally a third ramp of 7 °C/min to reach 230 °C, which was held for 5 min. Ion source and quadrupole temperatures were set at 230 °C and 150 °C, respectively. Full-scan mass spectra were acquired in the range of 30–350 *m/z* at a scan rate of 0.2 scans/s over a total run time of 66 min.

After sample extraction via HS-SPME, the fiber was thermally desorbed directly into the GC injector at 250 °C for 5 min to release the adsorbed volatile compounds prior to chromatographic separation.

### 4.4. Enantiomeric Analysis

Enantiomeric profiling was carried out using a chiral capillary column MEGA-DEX-DAC Beta (Mega, MI, Italy), composed of 2,3-diethyl-6-tert-butyldimethylsilyl-β-cyclodextrin as the chiral selector. The column had an internal diameter of 0.25 mm, a phase thickness of 0.25 µm, and a length of 25 m. It was installed on the same GC–MS system used for qualitative analysis. The sample amount, injector temperature, transfer line temperature, and MS parameters remained consistent with those used in the qualitative analysis. The split ratio was adjusted to 20:1. The GC method included an initial oven temperature of 60 °C held for 2 min, followed by a ramp of 2 °C/min until reaching 220 °C, which was maintained for an additional 2 min. To enable accurate compound identification and enantiomeric assignment, a homologous series of n-alkanes (C9–C25) was injected under identical conditions to calculate linear retention indices [43].

### 4.5. Identification of Volatile Compounds

The volatile compounds isolated from *L. mutica* were identified using a combination of Linear Retention Index (LRI) and mass spectra matching. LRI were calculated based on a co-injected series of n-alkanes (C9–C24; ChemService, West Chester, PA, USA) following the methodology described by Calva et al. [43]. Mass spectra were compared with NIST [44] and Adams [20] libraries and further confirmed by comparison with LRI values reported from the literature.

### 4.6. Data Analysis

Principal Coordinate Analysis (PCoA) based on the Bray–Curtis distance index was employed to visualize the chemical compounds of *L. mutica* (Benth.) in different parts of plants and seasons. In order to show the relationship between chemical compounds of *L. mutica* (response group) and parts of plants (explanatory group) in different seasons.

To test whether the two parts of plants (leaves and flowers) of *L. mutica* (Benth.) had significantly different compositions of chemical compound and to detect the effects of parts of plants and season variability, we performed a two-factor permutational multivariate analysis of variance (PERMANOVA) on the chemical composition data [45]. We used the Bray–Curtis distance measure and 999 random permutations. To assess compounds similarity among the different parts of plants and season, we performed additional pairwise PERMANOVA tests [46].

### 4.7. Statistical Analysis

Statistical analysis was performed to evaluate the consistency of the SPME extractions, with each sample analyzed in triplicate using independent fibers. These replicates were subjected to various statistical procedures as described by Hammer [47].

All statistical analyses were carried out using the software PAST version 5.0 (Paleontological Statistics), which provided visual and numerical outputs for data exploration and pattern recognition [42].

## 5. Conclusions

Chemical characterization of the *L. mutica* volatile compounds using HS-SPME/GC-MS technique revelated a complex and seasonally dynamic profile with significant seasonal variations during winter and summer. These findings offer valuable insights for optimizing the use of *L. mutica* in the pharmaceutical and food industries based on target compounds. PCA confirmed strong separation by plant part and season, underscoring the influence of environmental and phenological factors on secondary metabolism. The identification of enantiomerically pure (S)-(−)-α-pinene, (1*S*,3*R*)-(+)-δ-3-carene, and (S)-(+) linalool alongside racemic mixtures of other terpenes, highlights the critical role of chirality in its bioactivity potential. This work emphasizes the importance of considering phenological and climatic factors in phytochemical research. Future work will evaluate the biological activity of isolated enantiomers and assess the impact of altitude and soil on chemotype stability, advancing sustainable conservation and targeted utilization of this endemic Andean species.

## Figures and Tables

**Table 1:** Volatile compounds identified in different stages of *L. mutica* flowers development using SPME-GC-MS.

N°	Compounds	LRI ^a^	LRI ^b^	Winter			Summer		
				Feb	Mar	Apr	Jul	Aug	Sep
				% ± SD					
1	α-thujene	925	924	0.13 ± 0.01	0.23 ± 0.02	0.10 ± 0.01	n.d.	0.87 ± 0.08	0.19 ± 0.02
2	α-pinene	932	932	1.99 ± 0.19	3.68 ± 0.33	1.79 ± 0.18	2.36 ± 0.26	5.64 ± 0.51	3.41 ± 0.31
3	camphene	948	946	2.06 ± 0.20	3.66 ± 0.39	1.94 ± 0.19	2.61 ± 0.29	7.31 ± 0.70	4.13 ± 0.44
4	sabinene	971	969	0.01 ± 0.00	0.07 ± 0.01	0.03 ± 0.00	0.08 ± 0.01	0.05 ± 0.01	0.06 ± 0.01
5	β-pinene	977	974	0.43 ± 0.04	0.78 ± 0.07	0.39 ± 0.04	0.09 ± 0.01	n.d.	0.10 ± 0.01
6	myrcene	990	988	2.08 ± 0.23	4.62 ± 0.42	2.55 ± 0.28	2.28 ± 0.20	0.20 ± 0.02	0.09 ± 0.01
7	6-methyl-5-hepten-2-ol	992	989	0.01 ± 0.00	0.06 ± 0.01	0.05 ± 0.00	n.d.	n.d.	n.d.
8	δ-3-carene	1008	1008	0.33 ± 0.03	5.78 ± 0.55	3.15 ± 0.35	4.31 ± 0.48	7.25 ± 0.66	5.95 ± 0.54
9	α-terpinene	1018	1014	0.01 ± 0.00	0.11 ± 0.01	0.05 ± 0.01	0.01 ± 0.00	n.d.	n.d.
10	sylvestrene	1026	1025	0.01 ± 0.00	0.01 ± 0.00	0.01 ± 0.00	n.d.	n.d.	n.d.
11	ο-cymene	1029	1022	0.33 ± 0.03	0.41 ± 0.04	0.63 ± 0.06	0.34 ± 0.03	n.d.	0.38 ± 0.04
12	β-phellandrene	1036	1025	10.19 ± 0.92	7.81 ± 0.83	15.48 ± 1.47	17.74 ± 1.70	12.64 ± 1.15	17.12 ± 1.81
13	(*Z*)-β-ocimene	1045	1032	0.02 ± 0.00	0.08 ± 0.01	9.19 ± 0.88	0.06 ± 0.01	0.04 ± 0.00	0.03 ± 0.00
14	γ-terpinene	1051	1054	0.14 ± 0.01	0.07 ± 0.01	0.04 ± 0.00	0.59 ± 0.06	0.41 ± 0.04	0.33 ± 0.03
15	cis-linalool oxide	1076	1067	n.d.	n.d.	n.d.	0.60 ± 0.06	0.00 ± 0.00	0.03 ± 0.00
16	2-acetyl-3-methyl-pyrazine	1083	1079	0.04 ± 0.00	0.08 ± 0.01	n.d.	0.37 ± 0.04	0.12 ± 0.01	0.31 ± 0.03
17	ρ-mentha-2,4(8)-diene	1084	1085	0.03 ± 0.00	0.14 ± 0.01	0.14 ± 0.01	n.d.	0.21 ± 0.02	0.20 ± 0.02
18	terpinolene	1089	1086	0.08 ± 0.01	0.06 ± 0.01	0.05 ± 0.00	0.09 ± 0.01	0.12 ± 0.01	0.08 ± 0.01
19	δ-3-carene	1108	1007	0.71 ± 0.07	1.40 ± 0.13	1.12 ± 0.10	0.33 ± 0.04	3.34 ± 0.30	0.22 ± 0.02
20	1,3,8-ρ-menthatriene	1111	1108	0.07 ± 0.01	0.01 ± 0.00	0.07 ± 0.01	1.99 ± 0.22	2.26 ± 0.20	0.01 ± 0.00
21	1-octen-3-yl acetate	1114	1008	0.04 ± 0.00	0.06 ± 0.01	0.05 ± 0.00	0.21 ± 0.02	0.31 ± 0.03	0.31 ± 0.03
22	myrcenol	1117	1119	0.04 ± 0.00	0.01 ± 0.00	0.07 ± 0.01	n.d.	0.08 ± 0.01	0.10 ± 0.01
23	3-octanol acetate	1125	1120	0.02 ± 0.00	0.01 ± 0.00	0.03 ± 0.00	n.d.	0.55 ± 0.06	0.58 ± 0.05
24	iso-3-thujanol	1134	1134	0.69 ± 0.07	1.14 ± 0.12	1.16 ± 0.10	n.d.	n.d.	0.06 ± 0.01
25	cryptone	1184	1183	0.01 ± 0.00	0.07 ± 0.01	0.07 ± 0.01	n.d.	n.d.	0.07 ± 0.01
26	isoamyl tiglate	1191	1191	1.95 ± 0.18	0.03 ± 0.00	0.03 ± 0.01	n.d.	n.d.	0.03 ± 0.00
27	linalool formate	1211	1214	0.06 ± 0.01	0.10 ± 0.01	0.10 ± 0.01	0.06 ± 0.01	0.06 ± 0.01	0.07 ± 0.01
28	trans-carveol	1216	1215	0.01 ± 0.00	0.01 ± 0.00	n.d.	n.d.	n.d.	n.d.
29	cis-sabinene hydrate acetate	1220	1219	0.01 ± 0.00	0.01 ± 0.00	0.01 ± 0.00	n.d.	n.d.	n.d.
30	cis-carveol	1225	1226	0.01 ± 0.00	0.02 ± 0.00	0.03 ± 0.00	n.d.	n.d.	n.d.
31	pulegone	1232	1233	0.045 ± 0.005	0.09 ± 0.01	0.08 ± 0.01	0.04 ± 0.00	0.05 ± 0.01	0.04 ± 0.00
32	linalool acetate	1255	1254	0.01 ± 0.00	0.01 ± 0.00	0.01 ± 0.00	0.03 ± 0.00	n.d.	0.02 ± 0.00
33	bornyl acetate	1292	1284	0.01 ± 0.00	0.01 ± 0.00	0.02 ± 0.00	0.02 ± 0.00	n.d.	0.03 ± 0.00
34	2-undecanone	1299	1293	0.02 ± 0.00	0.03 ± 0.00	0.03 ± 0.00	n.d.	n.d.	0.24 ± 0.02
35	n-tridecane	1301	1300	0.01 ± 0.00	0.03 ± 0.00	0.01 ± 0.00	n.d.	n.d.	n.d.
36	δ-elemene	1337	1335	0.01 ± 0.00	0.01 ± 0.00	0.01 ± 0.00	n.d.	n.d.	n.d.
37	α-cubebene	1351	1348	0.01 ± 0.00	0.02 ± 0.00	0.01 ± 0.00	n.d.	n.d.	0.03 ± 0.00
38	cyclosativene	1364	1369	0.01 ± 0.00	0.01 ± 0.00	0.01 ± 0.00	n.d.	n.d.	0.03 ± 0.00
39	isoledene	1376	1374	0.62 ± 0.06	1.13 ± 0.10	0.98 ± 0.11	0.18 ± 0.02	0.20 ± 0.02	0.23 ± 0.02
40	α-copaene	1381	1374	0.01 ± 0.00	0.12 ± 0.01	0.12 ± 0.01	0.16 ± 0.02	0.14 ± 0.01	0.17 ± 0.02
41	α-duprezianene	1389	1387	0.01 ± 0.00	0.03 ± 0.00	0.03 ± 0.00	0.07 ± 0.01	n.d.	0.10 ± 0.01
42	7-epi-sesquithujene	1392	1390	0.01 ± 0.00	0.11 ± 0.01	0.10 ± 0.01	n.d.	n.d.	n.d.
43	cyperene	1396	1398	0.13 ± 0.01	0.22 ± 0.02	0.19 ± 0.02	0.07 ± 0.01	0.06 ± 0.01	0.09 ± 0.01
44	sesquithujene	1407	1405	0.26 ± 0.03	0.42 ± 0.04	0.37 ± 0.03	0.31 ± 0.03	0.14 ± 0.01	0.29 ± 0.03
45	α-gurjunene	1411	1409	0.64 ± 0.07	1.56 ± 0.15	1.39 ± 0.14	9.50 ± 0.86	8.87 ± 0.99	14.61 ± 1.39
46	α-cedrene	1416	1410	0.40 ± 0.04	0.72 ± 0.08	0.63 ± 0.06	0.32 ± 0.03	0.31 ± 0.03	0.39 ± 0.04
47	β-duprezianene	1420	1421	0.35 ± 0.04	0.76 ± 0.07	0.58 ± 0.06	1.48 ± 0.15	1.54 ± 0.14	0.20 ± 0.02
48	dictamnol	1429	1428	31.89 ± 3.05	12.91 ± 1.37	4.13 ± 0.45	4.54 ± 0.50	15.10 ± 1.51	3.57 ± 0.36
49	β-copaene	1434	1430	n.d.	n.d.	n.d.	0.59 ± 0.06	0.48 ± 0.05	0.64 ± 0.07
50	γ-elemene	1437	1434	2.93 ± 0.32	3.58 ± 0.36	3.22 ± 0.30	4.03 ± 0.44	1.26 ± 0.12	2.64 ± 0.25
51	6,9-guaiadiene	1441	1442	0.88 ± 0.08	2.02 ± 0.20	1.87 ± 0.19	0.58 ± 0.06	0.49 ± 0.05	0.66 ± 0.07
52	aromadendrene	1445	1439	1.02 ± 0.10	1.85 ± 0.19	1.75 ± 0.18	4.59 ± 0.50	4.17 ± 0.42	5.26 ± 0.53
53	neryl propanoate	1457	1452	0.28 ± 0.03	0.44 ± 0.04	0.45 ± 0.05	0.23 ± 0.02	0.15 ± 0.02	0.23 ± 0.02
54	α-acoradiene	1466	1464	1.02 ± 0.09	0.23 ± 0.02	0.21 ± 0.02	2.07 ± 0.23	2.35 ± 0.21	0.42 ± 0.04
55	9-epi-(*E*)-caryophyllene	1468	1464	6.42 ± 0.64	3.93 ± 0.43	6.18 ± 0.56	14.16 ± 1.42	14.37 ± 1.58	14.16 ± 1.27
56	β-acoradiene	1471	1469	0.14 ± 0.01	0.68 ± 0.07	0.66 ± 0.06	1.98 ± 0.22	0.85 ± 0.08	1.48 ± 0.15
57	10-epi-β-acoradiene	1474	1474	0.18 ± 0.02	0.28 ± 0.03	0.25 ± 0.02	0.44 ± 0.05	0.47 ± 0.04	0.66 ± 0.07
58	α-neocallitropsene	1476	1474	0.54 ± 0.06	0.91 ± 0.09	0.84 ± 0.08	2.79 ± 0.31	1.08 ± 0.11	2.04 ± 0.20
59	γ-gurjunene	1482	1475	0.36 ± 0.04	0.31 ± 0.03	0.32 ± 0.03	1.20 ± 0.13	1.06 ± 0.12	1.53 ± 0.16
60	γ-curcumene	1486	1481	1.82 ± 0.18	3.41 ± 0.34	3.73 ± 0.37	n.d.	n.d.	0.74 ± 0.08
61	cis-β-guaiene	1492	1492	1.50 ± 0.14	1.12 ± 0.12	1.47 ± 0.15	n.d.	n.d.	n.d.
62	neryl isobutanoate	1494	1490	0.10 ± 0.01	0.29 ± 0.03	0.21 ± 0.02	n.d.	n.d.	n.d.
63	bicyclogermacrene	1500	1500	7.00 ± 0.70	9.45 ± 0.95	9.74 ± 0.97	5.44 ± 0.54	2.40 ± 0.24	4.57 ± 0.46
64	trans-β-guaiene	1502	1502	0.92 ± 0.09	0.73 ± 0.07	0.52 ± 0.05	0.01 ± 0.00	0.01 ± 0.00	0.01 ± 0.00
65	allyl anthranilate	1504	1501	0.01 ± 0.00	0.40 ± 0.04	0.33 ± 0.03	n.d.	n.d.	n.d.
66	α-chamigrene	1507	1503	0.01 ± 0.00	0.01 ± 0.00	n.d.	n.d.	n.d.	n.d.
67	(*E*,*E*)-α-farnesene	1509	1505	5.02 ± 0.50	5.84 ± 0.64	7.62 ± 0.76	1.13 ± 0.11	0.56 ± 0.06	1.00 ± 0.10
68	β-curcumene	1516	1514	0.02 ± 0.00	0.00 ± 0.00	4.50 ± 0.45	0.80 ± 0.08	0.05 ± 0.01	0.58 ± 0.06
69	6-methyl-α-ionone	1519	1520	0.02 ± 0.00	2.15 ± 0.22	1.39 ± 0.14	n.d.	n.d.	0.04 ± 0.00
70	β-curcumene	1520	1514	0.54 ± 0.05	1.12 ± 0.11	1.09 ± 0.11	0.01 ± 0.00	0.01 ± 0.00	0.01 ± 0.00
71	1-phenyl heptan-3-one	1526	1524	0.03 ± 0.00	0.05 ± 0.01	0.05 ± 0.01	n.d.	n.d.	n.d.
72	δ-cadinene	1530	1522	0.06 ± 0.01	0.11 ± 0.01	0.12 ± 0.01	0.10 ± 0.01	0.31 ± 0.03	0.45 ± 0.05
73	(*Z*)-nerolidol	1535	1531	0.12 ± 0.01	0.19 ± 0.02	0.18 ± 0.02	0.13 ± 0.01	0.11 ± 0.01	0.14 ± 0.01
74	α-copaen-11-ol	1541	1539	0.01 ± 0.00	0.02 ± 0.00	0.02 ± 0.00	n.d.	n.d.	n.d.
75	silphiperfolan-6-β-ol	1544	1546	0.01 ± 0.00	0.03 ± 0.00	0.03 ± 0.00	n.d.	n.d.	n.d.
76	elemol	1551	1548	0.01 ± 0.00	0.02 ± 0.00	0.02 ± 0.00	n.d.	n.d.	n.d.
77	(*E*)-nerolidol	1562	1561	0.01 ± 0.00	0.00 ± 0.00	0.01 ± 0.00	n.d.	n.d.	n.d.
78	zierone	1576	1574	0.08 ± 0.01	0.11 ± 0.01	0.14 ± 0.01	n.d.	n.d.	n.d.
79	spathulenol	1579	1577	0.01 ± 0.00	0.01 ± 0.00	0.04 ± 0.00	0.06 ± 0.01	n.d.	0.04 ± 0.00
80	(*E*)-jasmolactone, extra c	1581	1578	0.12 ± 0.01	0.11 ± 0.01	0.20 ± 0.02	0.59 ± 0.06	1.01 ± 0.10	1.08 ± 0.11
81	β-copaen-4-α-ol	1587	1590	0.04 ± 0.00	0.04 ± 0.00	0.07 ± 0.01	0.25 ± 0.03	0.26 ± 0.03	0.30 ± 0.03
82	anti-anti-anti-helifolen-12-al B	1595	1592	0.17 ± 0.02	0.19 ± 0.02	0.29 ± 0.03	0.58 ± 0.06	0.85 ± 0.08	0.99 ± 0.10
83	cis-dihydro -mayurone	1599	1595	0.02 ± 0.00	0.37 ± 0.04	0.62 ± 0.06	n.d.	n.d.	0.02 ± 0.00
84	ledol	1603	1602	0.03 ± 0.00	0.02 ± 0.00	0.03 ± 0.00	n.d.	n.d.	n.d.
85	5-epi-7-epi-α-eudesmol	1606	1607	0.01 ± 0.00	0.02 ± 0.00	0.02 ± 0.00	n.d.	n.d.	n.d.
86	β-atlantol	1613	1608	0.03 ± 0.00	0.03 ± 0.00	0.05 ± 0.01	n.d.	n.d.	n.d.
87	junenol	1618	1618	0.02 ± 0.00	0.01 ± 0.00	0.01 ± 0.00	5.98 ± 0.60	0.00 ± 0.00	4.54 ± 0.45
88	selina-1,3,7(11)-trien-8-one	1630	1632	0.05 ± 0.01	0.05 ± 0.01	0.10 ± 0.01	n.d.	n.d.	n.d.
89	β-acorenol	1636	1636	0.01 ± 0.00	0.01 ± 0.00	n.d.	n.d.	n.d.	n.d.
90	cubenol	1645	1645	0.02 ± 0.00	0.02 ± 0.00	0.02 ± 0.00	n.d.	n.d.	0.02 ± 0.00
91	himachalol	1654	1652	0.05 ± 0.01	0.05 ± 0.01	0.07 ± 0.01	0.04 ± 0.00	0.07 ± 0.01	0.08 ± 0.01
92	α-cadinol	1659	1652	0.16 ± 0.02	0.17 ± 0.02	0.22 ± 0.02	0.18 ± 0.02	0.30 ± 0.03	0.28 ± 0.03
93	helifolenol A	1673	1674	0.75 ± 0.08	0.90 ± 0.09	1.09 ± 0.11	0.74 ± 0.07	1.30 ± 0.13	1.17 ± 0.12
94	helifolenol B	1679	1677	0.01 ± 0.00	0.01 ± 0.00	0.02 ± 0.00	n.d.	n.d.	0.04 ± 0.00
95	zizanal	1695	1697	0.01 ± 0.00	0.01 ± 0.00	0.01 ± 0.00	n.d.	n.d.	0.02 ± 0.00
96	amorpha-4,9-dien-2-ol	1699	1700	0.01 ± 0.00	0.01 ± 0.00	0.01 ± 0.00	n.d.	n.d.	n.d.
97	cembrene	1939	1937	0.01 ± 0.00	0.01 ± 0.00	0.01 ± 0.00	n.d.	n.d.	0.02 ± 0.00
98	(3*Z*)-cembrene A	1963	1965	0.01 ± 0.00	0.01 ± 0.00	0.01 ± 0.00	n.d.	n.d.	n.d.
99	nootkatinol	2087	2088	0.01 ± 0.00	0.01 ± 0.00	0.01 ± 0.00	n.d.	n.d.	n.d.
100	benzyl cinnamate	2100	2092	0.01 ± 0.00	0.01 ± 0.00	0.01 ± 0.00	n.d.	n.d.	n.d.
101	2-keto-manool oxide	2210	2216	n.d.	n.d.	n.d.	0.02 ± 0.00	0.01 ± 0.00	0.02 ± 0.00
monoterpenes hydrocarbons				17.69 ± 0.00	27.32 ± 0.00	35.35 ± 0.00	32.45 ± 0.00	37 ± 0.00	31.96 ± 0.00
oxygenated monoterpenes				3.6 ± 0.00	2.98 ± 0.00	2.87 ± 0.00	1.18 ± 0.00	4.34 ± 0.00	1.38 ± 0.00
sesquiterpenes hydrocarbons				54.9 ± 0.00	45.13 ± 0.00	42.52 ± 0.00	51.97 ± 0.00	41.15 ± 0.00	52.92 ± 0.00
oxygenated sesquiterpenes				7.97 ± 0.00	5.84 ± 0.00	8.64 ± 0.00	8.5 ± 0.00	3.9 ± 0.00	8.64 ± 0.00
others				4.25 ± 0.12	8.84 ± 0.41	7.59 ± 0.23	5.36 ± 0.31	15.43 ± 0.00	4.82 ± 0.00

^a^ Calculated linear retention index; ^b^ linear retention index according to [20]; n.d. = not detectable; % ± SD = percentage with standard deviation; values are the mean of three determinations; mean ± SD (*n* = 3).

**Table 2:** Volatile compounds identified in different stages of *L. mutica* leaves development using SPME-GC-MS.

N°	Compounds	LRI ^a^	LRI ^b^	Winter			Summer		
				Feb	Mar	Apr	Jul	Aug	Sep
				% ± SD					
1	α-thujene	927	924	0.16 ± 0.02	0.14 ± 0.01	0.13 ± 0.01	n.d.	n.d.	n.d.
2	α-pinene	934	932	3.94 ± 0.39	3.28 ± 0.36	3.14 ± 0.30	5.99 ± 0.66	6.80 ± 0.75	5.17 ± 0.57
3	camphene	951	946	5.61 ± 0.59	4.42 ± 0.48	4.39 ± 0.48	10.31 ± 1.08	11.98 ± 1.12	9.06 ± 0.90
4	sabinene	973	969	0.03 ± 0.00	0.00 ± 0.00	0.00 ± 0.00	0.09 ± 0.01	0.15 ± 0.02	0.08 ± 0.01
5	β-pinene	977	974	0.79 ± 0.08	0.61 ± 0.06	0.62 ± 0.06	0.08 ± 0.01	0.09 ± 0.01	0.06 ± 0.01
6	myrcene	990	988	3.24 ± 0.35	2.95 ± 0.29	0.04 ± 0.00	1.46 ± 0.16	2.21 ± 0.24	1.67 ± 0.18
7	6-methyl-5-hepten-2-ol	992	989	0.07 ± 0.01	0.00 ± 0.00	0.12 ± 0.01	n.d.	n.d.	n.d.
8	2-hydroxy-3-methyl-methyl pentanoate	993	989	0.11 ± 0.01	n.d.	n.d.	n.d.	n.d.	n.d.
9	δ-3-carene	1004	1003	2.86 ± 0.29	2.62 ± 0.26	1.14 ± 0.11	11.51 ± 1.15	10.15 ± 1.02	9.86 ± 0.99
10	α-terpinene	1022	1014	n.d.	n.d.	n.d.	0.04 ± 0.00	0.09 ± 0.01	0.06 ± 0.01
11	ρ-cymene	1007	1007	1.00 ± 0.10	1.05 ± 0.11	0.00 ± 0.00	0.24 ± 0.02	0.57 ± 0.06	0.33 ± 0.03
12	sylvestrene	1026	1025	0.01 ± 0.00	0.01 ± 0.00	n.d.	n.d.	n.d.	n.d.
13	ο-cymene	1029	1022	0.27 ± 0.03	0.00 ± 0.00	0.29 ± 0.03	n.d.	n.d.	n.d.
14	(*Z*)-β-ocimene	1033	1032	29.24 ± 2.92	12.58 ± 1.26	15.32 ± 1.53	4.50 ± 0.45	12.82 ± 1.28	2.24 ± 0.02
15	(*E*)-β-ocimene	1045	1044	0.20 ± 0.02	0.12 ± 0.01	0.14 ± 0.01	0.06 ± 0.01	0.15 ± 0.02	0.06 ± 0.01
16	γ-terpinene	1054	1054	0.05 ± 0.01	0.05 ± 0.01	0.05 ± 0.01	0.10 ± 0.01	0.08 ± 0.01	0.12 ± 0.01
17	meta-tolualdehyde	1064	1064	0.00 ± 0.00	0.08 ± 0.01	0.10 ± 0.01	n.d.	n.d.	n.d.
18	2-acetyl-3-methyl-pyrazine	1081	1079	0.14 ± 0.01	0.09 ± 0.01	0.12 ± 0.01	0.15 ± 0.02	0.12 ± 0.01	0.19 ± 0.02
19	ρ-mentha-2,4(8)-diene	1083	1085	n.d.	n.d.	n.d.	0.44 ± 0.04	0.87 ± 0.09	0.44 ± 0.04
20	terpinolene	1087	1086	0.01 ± 0.00	0.01 ± 0.00	0.05 ± 0.01	0.20 ± 0.02	0.41 ± 0.04	0.22 ± 0.02
21	trans-sabinene hydrate	1092	1098	n.d.	n.d.	n.d.	0.05 ± 0.01	0.10 ± 0.01	0.05 ± 0.01
22	linalool	1112	1095	0.20 ± 0.02	0.15 ± 0.02	0.23 ± 0.02	0.13 ± 0.01	0.28 ± 0.03	0.19 ± 0.02
23	1,3,8-ρ-menthatriene	1114	1117	0.08 ± 0.01	0.05 ± 0.01	0.07 ± 0.01	0.04 ± 0.00	0.04 ± 0.00	0.04 ± 0.00
24	1-octen-3-yl acetate	1117	1110	0.94 ± 0.09	0.48 ± 0.05	0.73 ± 0.07	0.40 ± 0.04	0.73 ± 0.07	0.46 ± 0.05
25	trans-rose oxide	1119	1122	0.06 ± 0.01	0.05 ± 0.01	0.08 ± 0.01	0.09 ± 0.01	0.32 ± 0.03	0.17 ± 0.02
26	3-octanol acetate	1124	1120	n.d.	n.d.	n.d.	0.34 ± 0.03	0.61 ± 0.06	0.42 ± 0.04
27	(*E*)-tagetone	1135	1139	0.03 ± 0.00	0.02 ± 0.00	0.02 ± 0.00	0.04 ± 0.00	0.71 ± 0.07	0.05 ± 0.01
28	iso-3-thujanol	1138	1134	0.09 ± 0.01	0.07 ± 0.01	0.07 ± 0.01	n.d.	n.d.	n.d.
29	3-thujanol	1162	1164	0.27 ± 0.03	0.15 ± 0.02	0.04 ± 0.00	n.d.	n.d.	n.d.
30	cis-linalool oxide	1170	1170	0.01 ± 0.00	0.01 ± 0.00	0.01 ± 0.00	n.d.	n.d.	n.d.
31	cryptone	1188	1178	0.08 ± 0.01	0.05 ± 0.01	0.10 ± 0.01	0.10 ± 0.01	0.14 ± 0.01	0.14 ± 0.01
32	myrtenol	1194	1194	0.05 ± 0.01	0.05 ± 0.01	0.00 ± 0.00	n.d.	n.d.	n.d.
33	2-methyoxythiophenol	1207	1210	0.01 ± 0.00	0.01 ± 0.00	0.07 ± 0.01	n.d.	n.d.	n.d.
34	linalool formate	1211	1214	0.06 ± 0.01	0.06 ± 0.01	0.01 ± 0.00	0.06 ± 0.01	0.14 ± 0.01	0.08 ± 0.01
35	cis-sabinene hydrate acetate	1220	1219	0.01 ± 0.00	0.01 ± 0.00	0.01 ± 0.00	n.d.	n.d.	n.d.
36	cis-carveol	1225	1226	0.03 ± 0.00	0.02 ± 0.00	0.02 ± 0.00	n.d.	n.d.	n.d.
37	pulegone	1235	1233	0.10 ± 0.01	0.06 ± 0.01	0.10 ± 0.01	0.07 ± 0.01	0.12 ± 0.01	0.08 ± 0.01
38	trans-sabinene hydrate acetate	1260	1253	0.01 ± 0.00	0.01 ± 0.00	0.02 ± 0.00	n.d.	n.d.	n.d.
39	3-thujanol acetate	1295	1295	0.06 ± 0.01	0.05 ± 0.01	0.05 ± 0.01	0.03 ± 0.01	0.05 ± 0.01	0.05 ± 0.01
40	cis-pinocarvyl acetate	1314	1311	0.01 ± 0.00	0.01 ± 0.00	0.01 ± 0.00	n.d.	n.d.	n.d.
41	δ-elemene	1337	1335	0.01 ± 0.00	0.01 ± 0.00	0.01 ± 0.00	n.d.	n.d.	n.d.
42	α-cubebene	1352	1348	0.01 ± 0.00	0.01 ± 0.00	0.01 ± 0.00	n.d.	n.d.	n.d.
43	NI	1358	--	0.02 ± 0.00	0.02 ± 0.00	0.01 ± 0.00	n.d.	n.d.	n.d.
44	α-ylangene	1376	1373	1.48 ± 0.15	1.33 ± 0.13	1.63 ± 0.16	0.05 ± 0.01	0.06 ± 0.01	0.29 ± 0.03
45	α-copaene	1381	1374	0.08 ± 0.01	0.07 ± 0.01	0.07 ± 0.01	0.00 ± 0.00	0.09 ± 0.01	0.06 ± 0.01
46	2-epi-α-funebrene	1384	1380	0.02 ± 0.00	0.02 ± 0.00	0.02 ± 0.00	0.38 ± 0.04	0.16 ± 0.02	0.00 ± 0.00
47	β-elemene	1389	1389	0.03 ± 0.00	0.02 ± 0.00	0.02 ± 0.00	0.06 ± 0.01	0.00 ± 0.00	0.10 ± 0.01
48	7-epi-sesquithujene	1392	1390	0.05 ± 0.01	0.03 ± 0.00	0.04 ± 0.00	0.00 ± 0.00	0.56 ± 0.06	0.39 ± 0.04
49	cyperene	1399	1398	0.23 ± 0.02	0.20 ± 0.02	0.24 ± 0.02	n.d.	n.d.	n.d.
50	sibirene	1402	1400	0.01 ± 0.00	0.01 ± 0.00	0.02 ± 0.00	n.d.	n.d.	n.d.
51	β-longipinene	1402	1400	0.02 ± 0.00	0.02 ± 0.00	0.01 ± 0.00	n.d.	n.d.	n.d.
52	sesquithujene	1409	1405	0.14 ± 0.01	0.12 ± 0.01	0.14 ± 0.01	n.d.	n.d.	n.d.
53	β-isocomene	1411	1407	0.08 ± 0.01	0.09 ± 0.01	0.11 ± 0.01	n.d.	n.d.	n.d.
54	α-gurjunene	1414	1409	1.37 ± 0.14	1.18 ± 0.12	1.45 ± 0.15	6.37 ± 0.64	10.04 ± 1.00	6.27 ± 0.63
55	α-cedrene	1418	1410	0.67 ± 0.07	0.57 ± 0.06	0.71 ± 0.07	0.20 ± 0.02	0.45 ± 0.05	0.40 ± 0.04
56	trans-α-ambrinol	1422	1421	0.90 ± 0.09	0.00 ± 0.00	0.65 ± 0.07	0.93 ± 0.09	0.00 ± 0.00	1.03 ± 0.10
57	β-duprezianene	1425	1428	0.22 ± 0.02	0.85 ± 0.08	0.31 ± 0.03	0.30 ± 0.03	0.00 ± 0.00	0.00 ± 0.00
58	dictamnol	1437	1428	9.85 ± 0.99	34.86 ± 3.49	34.64 ± 3.46	11.05 ± 1.11	10.64 ± 1.06	15.15 ± 1.52
59	β-copaene	1440	1430	1.32 ± 0.13	1.25 ± 0.13	1.44 ± 0.14	0.90 ± 0.09	0.30 ± 0.03	1.41 ± 0.14
60	aromadendrene	1444	1439	2.55 ± 0.26	2.46 ± 0.25	2.88 ± 0.29	0.95 ± 0.09	1.48 ± 0.15	5.87 ± 0.59
61	neryl propanoate	1456	1452	2.11 ± 0.21	2.05 ± 0.20	2.59 ± 0.26	0.18 ± 0.02	0.00 ± 0.00	0.00 ± 0.00
62	α-patchoulene	1460	1454	0.20 ± 0.02	0.18 ± 0.02	0.22 ± 0.02	0.30 ± 0.03	0.56 ± 0.06	0.47 ± 0.05
63	NI	1461	1464	0.32 ± 0.03	0.33 ± 0.03	0.36 ± 0.04	n.d.	n.d.	n.d.
64	9-epi-(*E*)-caryophyllene	1467	1466	3.59 ± 0.36	4.83 ± 0.48	5.96 ± 0.60	7.64 ± 0.76	9.48 ± 0.95	10.64 ± 1.06
65	γ-gurjunene	1470	1475	0.83 ± 0.08	0.80 ± 0.08	0.93 ± 0.09	0.38 ± 0.04	0.41 ± 0.04	0.38 ± 0.04
66	γ-curcumene	1474	1481	0.12 ± 0.01	0.12 ± 0.01	0.13 ± 0.01	1.52 ± 0.15	1.72 ± 0.17	1.38 ± 0.14
67	11-α-himachala-1,4-diene	1476	1485	0.46 ± 0.05	0.41 ± 0.04	0.48 ± 0.05	2.33 ± 0.23	1.86 ± 0.19	1.64 ± 0.16
68	neryl isobutanoate	1482	1490	0.37 ± 0.04	0.38 ± 0.04	0.43 ± 0.04	0.22 ± 0.02	0.27 ± 0.03	0.01 ± 0.00
69	α-zingiberene	1485	1493	0.72 ± 0.07	0.68 ± 0.07	0.71 ± 0.07	2.54 ± 0.25	1.48 ± 0.15	2.00 ± 0.20
70	bicyclogermacrene	1492	1500	0.50 ± 0.05	0.47 ± 0.05	0.61 ± 0.06	0.17 ± 0.02	0.31 ± 0.03	0.25 ± 0.03
71	α-zingiberene	1499	1493	6.23 ± 0.62	6.66 ± 0.67	0.00 ± 0.00	n.d.	n.d.	n.d.
72	trans-β-guaiene	1502	1502	3.23 ± 0.32	2.58 ± 0.26	3.52 ± 0.35	4.68 ± 0.47	3.68 ± 0.37	4.95 ± 0.50
73	(E,E)-α-farnesene	1504	1505	0.19 ± 0.02	0.17 ± 0.02	0.22 ± 0.02	0.58 ± 0.06	0.40 ± 0.04	0.50 ± 0.05
74	β-curcumene	1515	1514	9.64 ± 0.96	4.40 ± 0.44	8.76 ± 0.88	1.40 ± 0.14	1.10 ± 0.11	0.84 ± 0.08
75	6-methyl-α-ionone	1523	1520	0.04 ± 0.00	0.03 ± 0.00	0.04 ± 0.00	0.04 ± 0.00	0.00 ± 0.00	0.00 ± 0.00
76	1-phenyl heptan-3-one	1525	1524	0.03 ± 0.00	0.03 ± 0.00	0.04 ± 0.00	n.d.	n.d.	n.d.
77	δ-cadinene	1529	1522	0.07 ± 0.01	0.06 ± 0.01	0.07 ± 0.01	0.23 ± 0.02	0.14 ± 0.01	0.17 ± 0.02
78	γ-vetivenene	1532	1531	0.05 ± 0.01	0.07 ± 0.01	0.07 ± 0.01	n.d.	n.d.	n.d.
79	(Z)-nerolidol	1535	1531	0.06 ± 0.01	0.05 ± 0.01	0.07 ± 0.01	0.14 ± 0.01	0.07 ± 0.01	0.08 ± 0.01
80	α-copaen-11-ol	1541	1539	0.01 ± 0.00	0.01 ± 0.00	0.01 ± 0.00	n.d.	n.d.	n.d.
81	selina-3,7(11)-diene	1544	1545	0.02 ± 0.00	0.02 ± 0.00	0.02 ± 0.00	0.04 ± 0.00	0.00 ± 0.00	0.00 ± 0.00
82	zierone	1575	1574	0.14 ± 0.01	0.11 ± 0.01	0.13 ± 0.01	0.66 ± 0.07	0.08 ± 0.01	0.00 ± 0.00
83	(*E*)-jasmolactone, extra C	1580	1578	0.05 ± 0.01	0.09 ± 0.01	0.10 ± 0.01	0.05 ± 0.01	0.00 ± 0.00	0.00 ± 0.00
84	thujopsan-2-α-ol	1587	1586	0.07 ± 0.01	0.11 ± 0.01	0.10 ± 0.01	1.81 ± 0.18	1.05 ± 0.11	0.85 ± 0.09
85	β-copaen-4-α-ol	1592	1590	0.01 ± 0.00	0.02 ± 0.00	0.00 ± 0.00	0.93 ± 0.09	0.52 ± 0.05	0.59 ± 0.06
86	cis-dihydro -mayurone	1595	1595	0.49 ± 0.05	0.80 ± 0.08	0.79 ± 0.08	0.99 ± 0.10	0.44 ± 0.04	0.48 ± 0.05
87	9,11-epoxy-guaia-3,10(14)-diene	1599	1601	0.35 ± 0.04	0.59 ± 0.06	0.00 ± 0.00	0.05 ± 0.01	0.00 ± 0.00	0.00 ± 0.00
88	ledol	1603	1602	0.05 ± 0.01	0.07 ± 0.01	0.07 ± 0.01	n.d.	n.d.	n.d.
89	isolongifolan-7-α-ol	1613	1618	0.07 ± 0.01	0.08 ± 0.01	0.08 ± 0.01	0.12 ± 0.01	0.06 ± 0.01	0.06 ± 0.01
90	junenol	1630	1618	0.05 ± 0.01	0.09 ± 0.01	0.08 ± 0.01	9.19 ± 0.92	0.00 ± 0.00	11.68 ± 1.17
91	β-acorenol	1636	1636	n.d.	n.d.	n.d.	0.03 ± 0.00	0.02 ± 0.00	0.01 ± 0.00
92	epi-α-cadinol	1645	1638	0.01 ± 0.00	0.02 ± 0.00	0.02 ± 0.00	0.05 ± 0.01	0.11 ± 0.01	0.12 ± 0.01
93	himachalol	1654	1652	0.03 ± 0.01	0.04 ± 0.01	0.05 ± 0.01	0.23 ± 0.02	0.00 ± 0.00	0.38 ± 0.04
94	α-cadinol	1659	1652	0.10 ± 0.01	0.13 ± 0.01	0.14 ± 0.01	0.86 ± 0.09	0.43 ± 0.04	0.00 ± 0.00
95	cis-calamenen-10-ol	1662	1660	n.d.	n.d.	n.d.	0.03 ± 0.00	0.00 ± 0.00	0.00 ± 0.00
96	helifolenol A	1673	1674	0.49 ± 0.05	0.60 ± 0.06	0.67 ± 0.07	4.50 ± 0.45	2.32 ± 0.23	1.88 ± 0.19
97	helifolenol B	1679	1677	0.01 ± 0.00	0.02 ± 0.00	0.00 ± 0.00	0.09 ± 0.01	0.00 ± 0.00	0.00 ± 0.00
98	amorpha-4,9-dien-2-ol	1698	1700	0.01 ± 0.00	0.02 ± 0.00	0.00 ± 0.00	n.d.	n.d.	n.d.
99	(3*Z*)-cembrene A	1963	1965	0.02 ± 0.00	0.01 ± 0.00	0.00 ± 0.00	n.d.	n.d.	n.d.
100	2-keto-manool oxide	2210	2216	n.d.	n.d.	n.d.	0.12 ± 0.01	0.08 ± 0.01	0.05 ± 0.01
monoterpenes hydrocarbons				47.40 ± 0.54	27.84 ± 0.14	25.30 ± 0.22	34.98 ± 0.43	46.32 ± 0.41	27.35 ± 0.19
oxygenated monoterpenes				1.75 ± 0.12	1.06 ± 0.09	1.29 ± 0.08	1.12 ± 0.11	2.87 ± 0.25	1.43 ± 0.16
sesquiterpenes hydrocarbons				43.49 ± 0.23	61.26 ± 0.51	63.12 ± 0.43	30.96 ± 0.22	34.27 ± 0.12	38.03 ± 0.41
oxygenated sesquiterpenes				13.84 ± 0.18	9.13 ± 0.10	13.84 ± 0.12	18.65 ± 0.21	4.64 ± 0.09	15.64 ± 0.08
others				2.22 ± 0.07	2.75 ± 0.09	2.06 ± 0.11	14.04 ± 0.13	11.98 ± 0.30	17.25 ± 0.19

^a^ Calculated linear retention index; ^b^ linear retention index according to [20]; n.d. = not detectable; % ± SD = percentage with standard deviation; values are the mean of three determinations; mean ± SD (*n* = 3).

**Table 3:** Results of two-factor PERMANOVA analysis of chemical composition of *L. mutica* by parts of plants and season.

Factor	df	SS	MS	Pseudo-F	R	*p*-Value
Plant parts	1	7713.9	7713.9	22.494	35.05	0.001
Season	1	5004.3	5004.3	14.593	27.873	0.002
Plant parts × Season	1	945.63	945.63	2.7575	14.174	0.074
Res	8	2743.5	342.93			
Total	11	16407				

**Table 4:** Results of the SIMPER analyses.

Chemical Compounds	Leaves	Flowers	Dissimilarity (%)	CD (%)
β-phellandrene	0	2.64	3.83	6.68
(Z)-β-ocimene	2.23	0.42	2.88	5.01
bicyclogermacrene	0	1.93	2.81	4.89
γ-elemene	0	1.34	1.95	3.4
aromadendrene	0	1.33	1.92	3.34
trans-β-guaiene	1.55	0.27	1.84	3.2
aromadendrene	1.23	0	1.79	3.12
(E,E)-α-farnesene	0.29	1.29	1.47	2.57
junenol	0.85	0.62	1.46	2.55
β-curcumene	1.43	0.66	1.39	2.41
dictamnol	2.88	2.3	1.29	2.25
α-gurjunene	1.49	1.62	1.28	2.23
α-neocallitropsene	0	0.81	1.17	2.04
δ-3-carene	1.79	1.56	1.17	2.04
α-zingiberene	0.81	0	1.17	2.03
11-α-himachala-1,4-diene	0.72	0	1.03	1.8
6,9-guaiadiene	0	0.69	1	1.74
camphene	2.09	1.46	1	1.73
δ-3-carene	0	0.69	1	1.73
α-zingiberene	0.67	0	0.99	1.73
γ-curcumene	0.52	0.77	0.96	1.67
α-acoradiene	0	0.63	0.92	1.61
neryl propanoate	0.62	0	0.92	1.61
β-acoradiene	0	0.63	0.9	1.57
myrcene	0.98	0.93	0.87	1.52
β-duprezianene	0	0.56	0.81	1.4
β-copaene	0.72	0.23	0.77	1.35
α-ylangene	0.51	0	0.77	1.34
9-epi-(E)-caryophyllene	2.03	2.29	0.75	1.31
cis-β-guaiene	0	0.43	0.63	1.1

CD: Contribution of each compound to the dissimilarity (%).

**Table 5:** Enantioselective analysis in flowers of *L. mutica* using β-cyclodextrin column.

Component	LRI ^a^	LRI ^b^	Enantiomeric Distribution	*e.e.* ± SD (%)
(1*R*)-(+)-α-pinene	936	932	71.29	42.58 ± 0.34
(1*S*)-(-)-α-pinene	937	932	28.71	
(*S*)-(-)-β-pinene	940	969	100	
(1*S*,3*R*)-(+)-δ-3-carene	1016	1008	100	
(*S*)-(+)-linalool	1107	1095	100	
(1*S*,4*S*,7*R*)-(+)-δ-cadinene	1418	1522	32.23	35.54 ± 0.27
(1*R*,4*R*,7*S*)-(–)-δ-cadinene	1430	1522	67.77	
(6*R*,8*S*)-(+)-α-muurolene	1498	1500	17.99	64.02 ± 0.66
(6*S*,8*R*)-(–)-α-muurolene	1502	1500	82.01	

LRI ^a^ = linear retention index calculated; LRI ^b^ = linear retention index bibliographic [21] *e.e.* = enantiomeric excess; mean ± SD (*n* = 3).

**Table 6:** Enantioselective analysis in leaves of *L. mutica* using β-cyclodextrin column.

Component	LRI ^a^	LRI ^b^	Enantiomeric Distribution	*e.e.* ± SD (%)
(1*R*)-(+)-α-pinene	927	932	89.83	79.66 ± 0.82
(1*S*)-(-)-α-pinene	937	932	10.17	
(*S*)-(-)-β-pinene	939	969	100	
(1*S*,3*R*)-(+)-δ-3-carene	982	1008	100	
(*S*)-(+)-linalool	1117	1095	100	
(1*S*,4*S*,7*R*)-(+)-δ-cadinene	1418	1522	25.31	49.38 ± 0.22
(1*R*,4*R*,7*S*)-(–)-δ-cadinene	1431	1522	74.69	
(6*R*,8*S*)-(+)-α-muurolene	1498	1500	51.20	1.59 ± 0.09
(6*S*,8*R*)-(–)-α-muurolene	1502	1500	49.60	

LRI ^a^ = linear retention index calculated; LRI ^b^ = linear retention index bibliographic [21] *e.e.* = enantiomeric excess; mean ± SD (*n* = 3).

## Data Availability

The original contributions presented in the study are included in the article; further inquiries can be directed to the corresponding author.

## Supplementary Materials

The following supporting information can be downloaded at: https://www.mdpi.com/article/10.3390/plants14193103/s1.
