# Supplementary material for: Seasonal Influence on Volatile Organic Compounds from Flowers and Leaves of Lepechinia mutica Extracted by SPME-GC-MS
Source: Plants (Basel). 2025 Oct 9;14(19):3103. doi: 10.3390/plants14193103 (PMC12525992; doi:10.3390/plants14193103)
Supplement: Supplementary file 1 [file plants-14-03103-s001.zip › plants-3880807-supplementary.pdf]

Supplementary Materials

Figure S1: Volatile compounds emitted by *Lepechinia mutica* flowers in February using SPME-GC analysis.

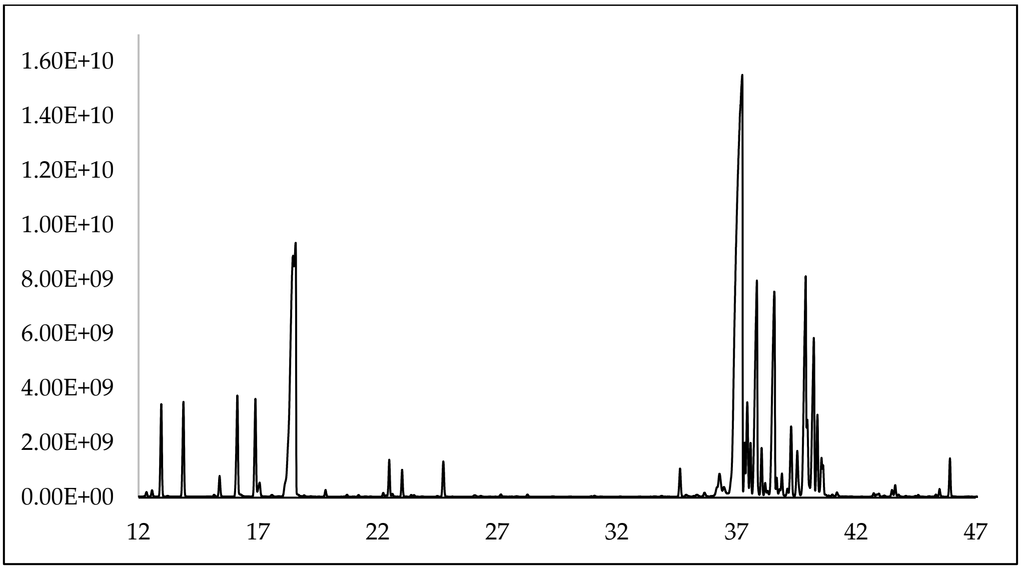

Figure S2: Volatile compounds emitted by *Lepechinia mutica* flowers in March using SPME-GC analysis.

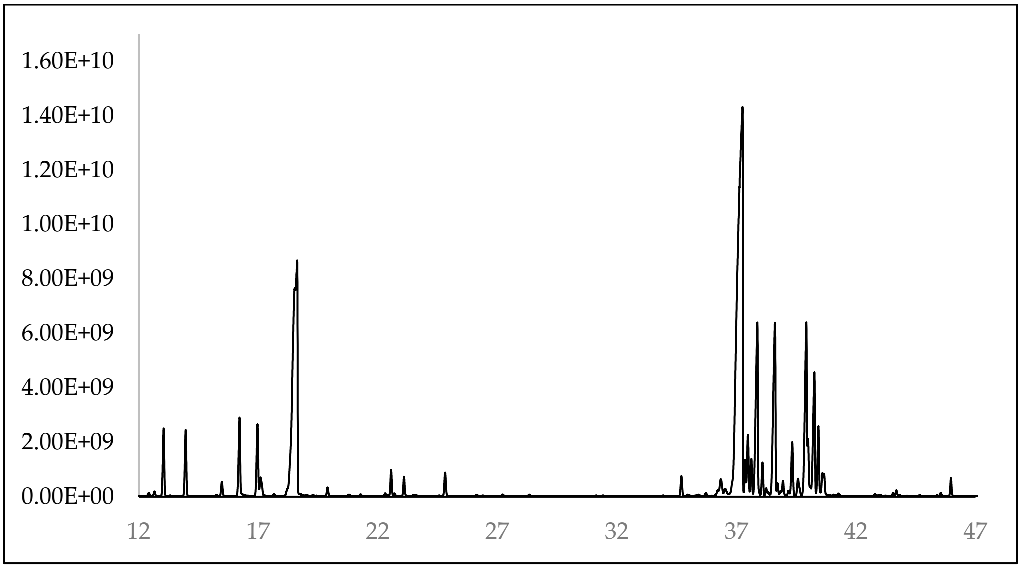

Figure S3: Volatile compounds emitted by *Lepechinia mutica* flowers in April using SPME-GC analysis.

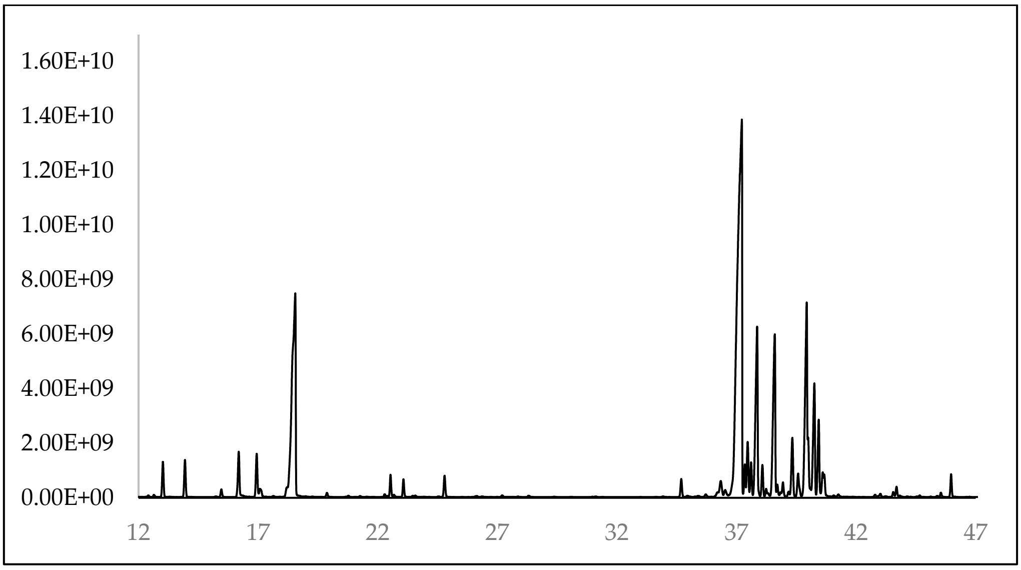

Figure S4: Volatile compounds emitted by *Lepechinia mutica* flowers in July using SPME-GC analysis.

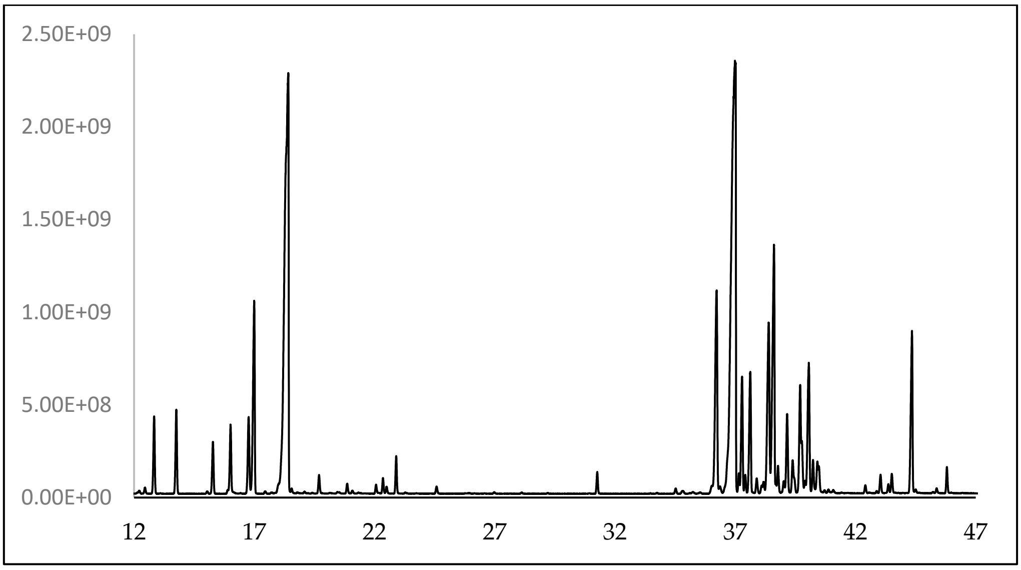

Figure S5: Volatile compounds emitted by *Lepechinia mutica* flowers in August using SPME-GC analysis.

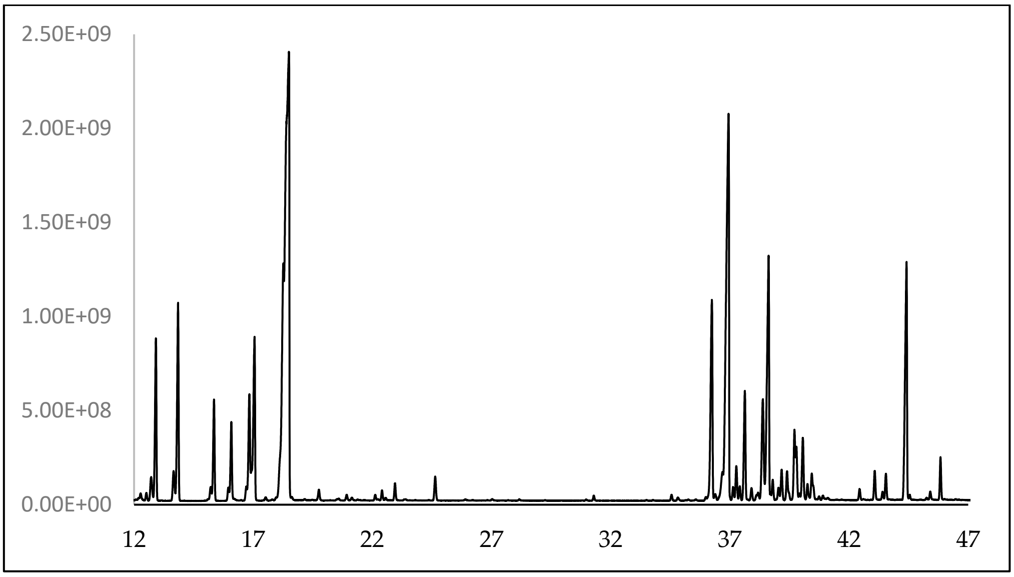

Figure S6: Volatile compounds emitted by *Lepechinia mutica* flowers in September using SPME-GC analysis.

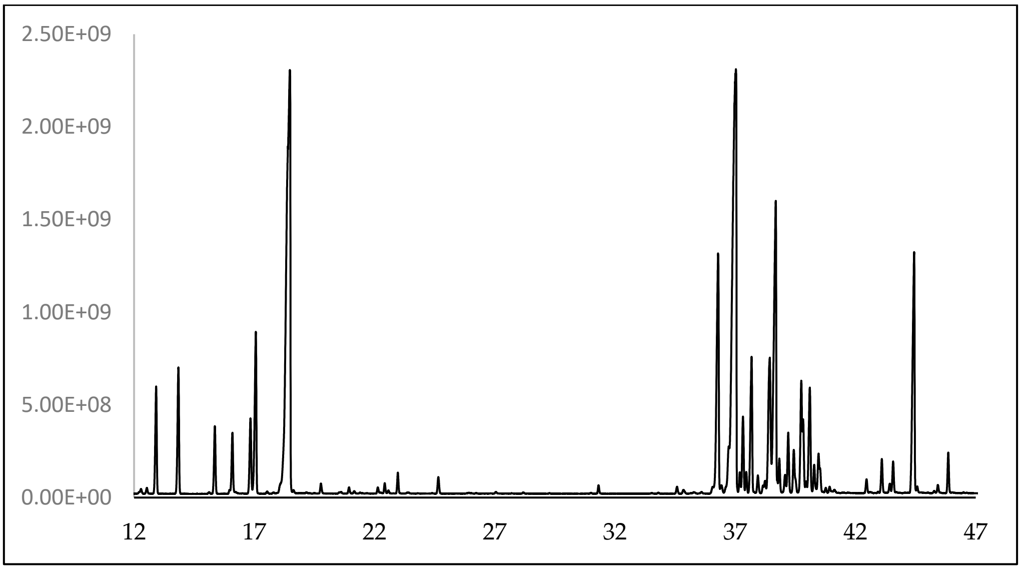

Figure S7: Volatile compounds emitted by *Lepechinia mutica* leaves in February using SPME-GC analysis.

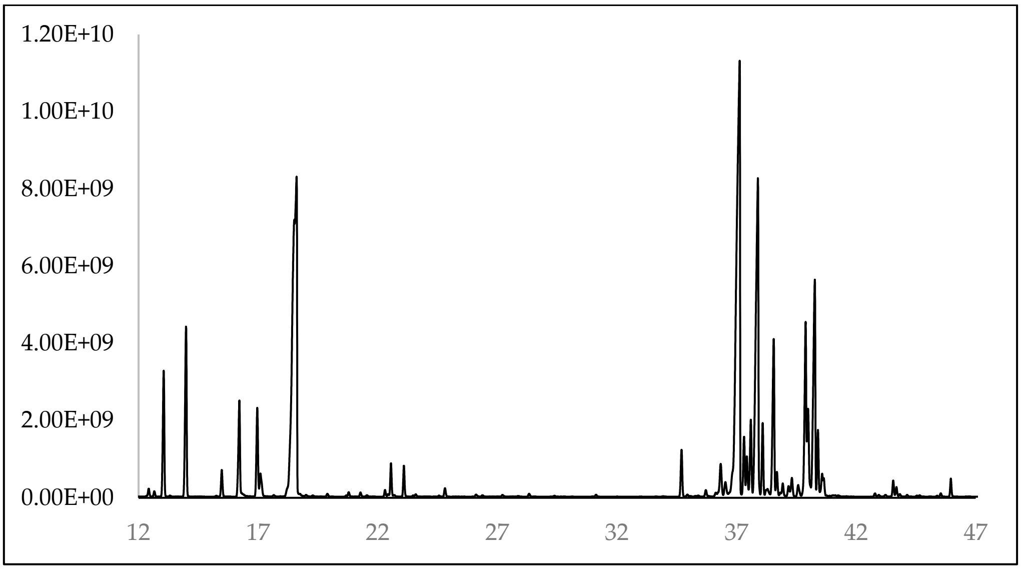

Figure S8: Volatile compounds emitted by *Lepechinia mutica* leaves in March using SPME-GC analysis.

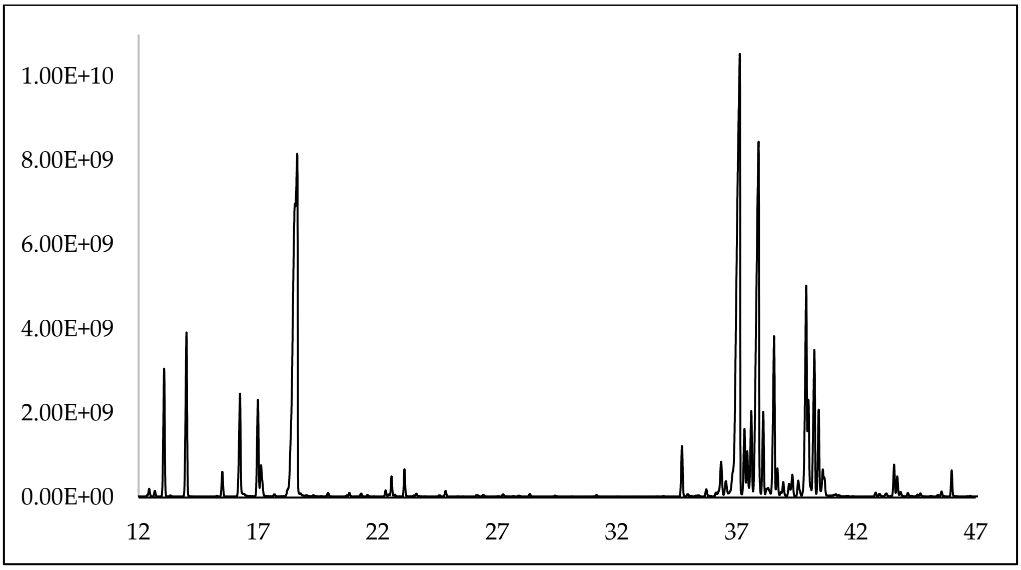

Figure S9: Volatile compounds emitted by *Lepechinia mutica* leaves in April using SPME-GC analysis.

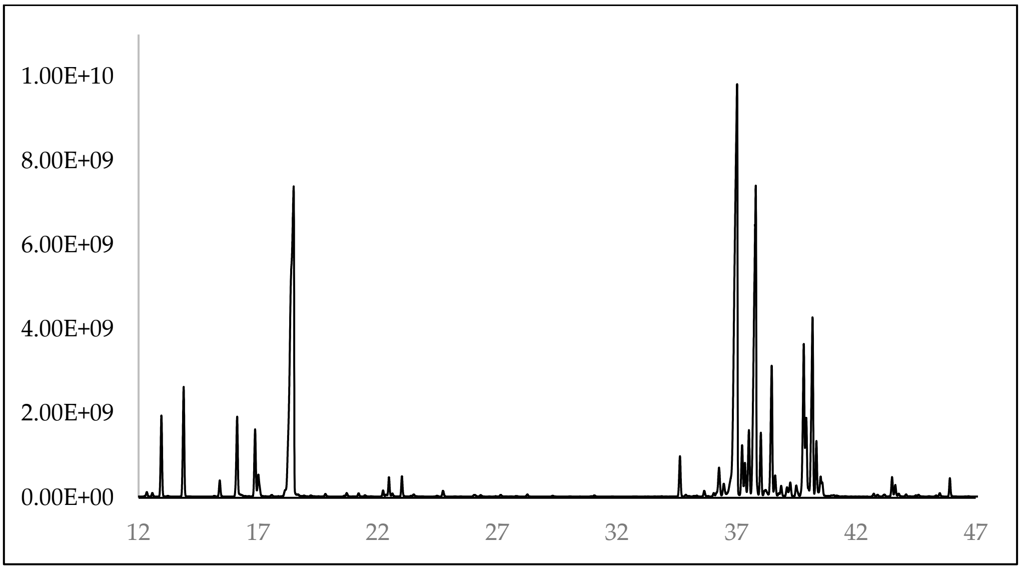

Figure S10: Volatile compounds emitted by *Lepechinia mutica* leaves in July using SPME-GC analysis.

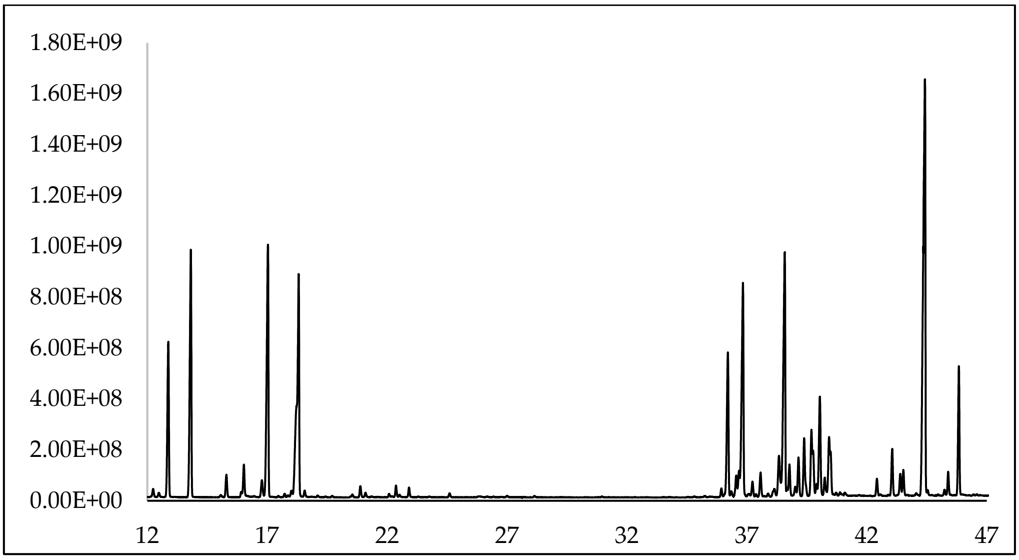

Figure S11: Volatile compounds emitted by *Lepechinia mutica* leaves in August using SPME-GC analysis.

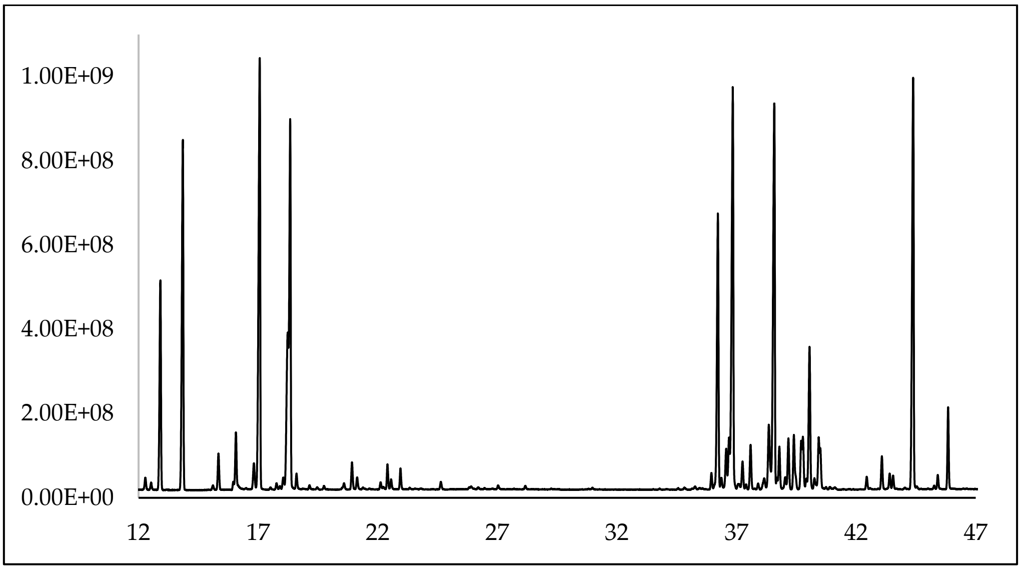

Figure S12: Volatile compounds emitted by *Lepechinia mutica* leaves in September using SPME-GC analysis.

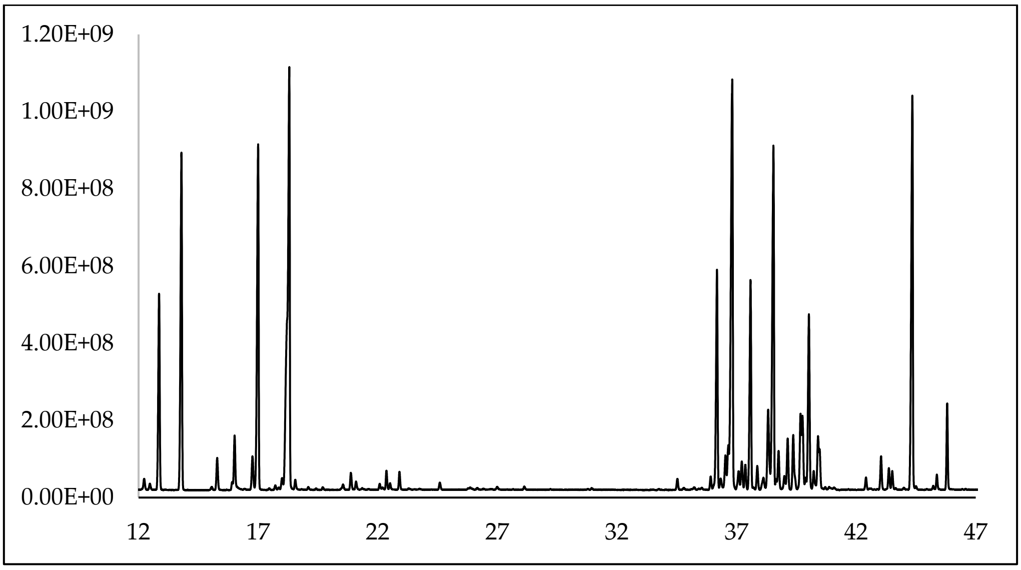

Figure S13: Chemical structure of majority compounds in flowers (a) and (b) and leaves (c) and (d)

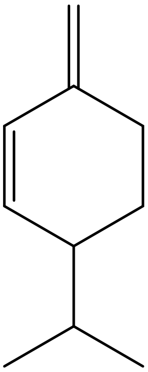

a) β-phellandrene

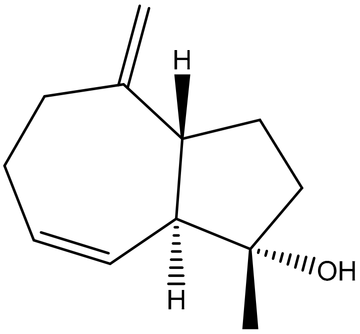

b) dictamnol

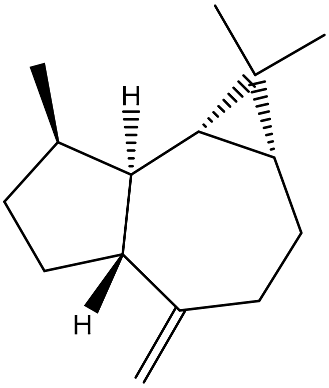

c) aromadendrene

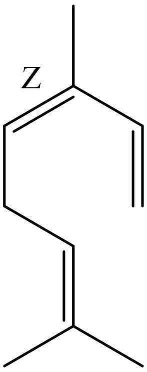

d) (Z)-β-ocimene
